# Early Workplace Intervention to Improve the Work Ability of Employees with Musculoskeletal Disorders in a German University Hospital—Results of a Pilot Study

**DOI:** 10.3390/healthcare4030064

**Published:** 2016-09-07

**Authors:** Monika Schwarze, Christoph Egen, Christoph Gutenbrunner, Stephanie Schriek

**Affiliations:** 1 Department for Rehabilitation Medicine, Hannover Medical School, Carl-Neuberg-Str. 1, Hannover 30625, Germany; egen.christoph@mh-hannover.de (C.E.); gutenbrunner.christoph@mh-hannover.de (C.G.); 2 Company Physician, Hannover Medical School, Carl-Neuberg-Str. 1, Hannover 30625, Germany; schriek.stephanie@mh-hannover.de

**Keywords:** work ability, musculoskeletal disorders, occupational health, worksite rehabilitation and prevention, sickness absence

## Abstract

Health promotion is becoming increasingly important in work life. Healthcare workers seem to be at special risk, experiencing musculoskeletal disorders (MSD); their situation is strongly influenced by demographic changes. The aim of this study is to evaluate the feasibility and outcome of a worksite intervention. In a one-group pretest-posttest design, 118 employees of a hospital were recruited from 2010 to 2011. The raised parameters were satisfaction with the program, work ability (Work Ability Index), and sickness absence (provided by human resource management). Patient-reported questionnaire data was raised at baseline (t1) and after three months (t2). Sickness leave was evaluated in the period six months prior to and six months after the intervention. Means, frequencies, standardized effect sizes (SES), analysis of variance, and regression analysis were carried out. Participants were found to be highly satisfied. Work ability increased with moderate effects (SES = 0.34; *p* < 0.001) and prognosis of gainful employment (SES = −0.19; *p* ≤ 0.047) with small effects. Days of MSD-related sickness absence were reduced by 38.5% after six months. The worksite intervention program is transferable to a hospital setting and integration in occupational health management is recommended. The use of a control group is necessary to demonstrate the effectiveness.

## 1. Introduction

Health promotion for employees to maintain work ability and participation in work life is becoming increasingly important against the background of demographic changes. Of particular relevance is to keep workers in the healthcare sector healthy and motivated until retirement age. In this sector it is anticipated that a higher number of people will need healthcare due to longer life expectancy, whilst alongside this a shortage of healthcare workers, especially in the nursing sector, is expected to exacerbate the issue [1].

These profound demographic changes are occurring in most industrialized nations, notably including those in Europe. In the context of work life they become apparent in the aging and the shrinkage of the labor force [2]. There are numerous implications associated with these changes in relation to politics, social security systems and companies [2]. In Germany, for example, the retirement age has been raised, thereby prolonging the working lifetime [3]. However, epidemiological studies show with increasing age, the prevalence of chronic disease rises and work ability decreases [4,5]. It is known that approximately one-third of the working population suffers from a chronic disease [6]. This goes along with higher sickness absence and early retirement, owing to an inability to remain at work, due to health problems [7]. Musculoskeletal disorders (MSDs) have a substantial impact on disability among adults of working age. They are also influenced by psychosocial factors in development and onset [8]. With a frequency of 53%, MSDs constitute the highest number of work-related disorders in Europe. They accounted for 49% of all sickness absences lasting two weeks or more and 60% of permanent incapacity cases. Total costs of MSDs were up to 2% of the gross domestic product (GDP) [9]. Compared with workers in other industrial sectors, healthcare workers rank high in incidence in most work-related complaints [10,11].

To encourage labor force participation, different actions have been implemented. The federal government has, for example, initiated a strategy characterized by the slogan “Every age counts”, which focuses on healthier, better-qualified employees and a longer work life [12]. In public and private companies, corporate health management and health promotion systems receive additional support and emphasis. As one process in Germany since 2004, Company Integration Management (BEM, Betriebliches Eingliederungs Management) is legally binding. Employers have to offer this tool for all employees who have been unable to work for more than six weeks within a year. The objective of this is to secure participation in employment [13].

In particular, early emphasis on prevention and treatment of MSDs preserves continued participation in gainful work [14,15,16]. A meta-analysis shows moderate evidence of interventions targeting the beginning symptoms of MSDs in the workplace to prevent work disability [17]. Furthermore, preventative and rehabilitative measures have evolved considerably in the direction of vocational orientation to preserve health, the ability to work and gainful employment. Basic components for successfully remaining and returning to work are a combination of early intervention, the cooperation of all stakeholders, multidisciplinary interventions and work-related measures [18,19,20]. It has been shown that stronger work-related rehabilitation has positive effects on vocational participation [21].

In Germany, in the field of medical and vocational rehabilitation carried out by the German pension insurance for legal reasons, the focus lies on participation in gainful working life [22]. Thus, concepts and interventions are largely workplace-orientated. Work-related medical rehabilitation (WMR) has gained importance in clinical practice and research in the last two decades [22,23,24,25,26,27]. Since 2009 the pension insurance has also had the opportunity to provide preventative treatment for policy-holders at high risk of losing earning capacity due to health reasons. Since then, various preventative pilot projects in workplace settings combined with existing structures of the pension insurance have been developed [16].

The concept of work ability used in this study is defined by the Finish Institute of Occupational Health as the balance between human resources and the demands of work [28]. The Work Ability Index (WAI) as an instrument and a tool was developed to identify, maintain and promote work ability. Evidence suggests that investments in active aging and promotion of work ability are worthwhile [28,29,30]. The WAI is also recommended by a German interdisciplinary working group to be used in projects on early interventions to maintain and restore work ability [31].

In our study we introduced a program to preserve the work ability of persons at risk of losing their earning capacity, but without high rehabilitation needs. The out-patient worksite intervention was introduced first in 2009 within the concept of the Job^Rehab^ model [32]. As a result, the health status and working capacity of employees with MSDs improved and a significant reduction of time lost due to sick leave was achieved [33]. Due to the program’s promising results in the industrial and service field, in the present study one element of the Job^Rehab^ model (Level I) was implemented for the first time for university employees in the healthcare field [34].

The program includes a three-month combined approach of workplace management, company medical service, and work-related out-patient medical prevention and rehabilitation.

In a pilot study, the feasibility and the outcome of this intervention were the focus. We investigated the employee’s satisfaction with the worksite program and examined changes in work ability, prognosis of gainful employment, and amount of sickness leave.

## 2. Materials and Methods

### 2.1. Study Design

The feasibility study was carried out in a prospective one-group pretest-posttest design. A control group was not established because of ethical reasons. To improve the lack of significance and enhance the meaningfulness of the results, the subjective prognosis of employment (SPE Scale) and regression analysis were added.

The intervention, a three-month work-related program was implemented at a university hospital, and workers were invited to participate. Workers completed questionnaires at baseline (prior to the intervention) and three months later (at the end of the intervention). The hospital’s human resources management made available data of the workers absenteeism prior and post to the intervention. The compared periods were six months before and six months after the intervention. The study was approved by the Ethic Commission at the Medical School Hannover, and all subjects signed an informed consent to participate.

### 2.2. Participants

A total of 645 workers from 11 selected divisions of the hospital were informed and invited. We recruited participants (*n* = 118) for the interventions from August 2010 to September 2011. Inclusion criteria were used in accordance with the Job^Rehab^ model [32,35]. The criteria included; MSD, work-related functional limitations, anticipated employment period in the company being at least one year and no intention of retirement. Employees with acute pain, incipient paralysis and severe nerve root irritation of the spine were excluded. Depending on the severity of the disease, functional impairments and duration of sick leave 12 months prior to the intervention, as well as different personal and environmental contextual factors, the company physician (CP) recommended the worksite intervention (corresponding to Level I) according to the Job^Rehab^ model [35]. Level I consisted of an intensive, one-week outpatient prevention program with rehabilitative elements. For a detailed description of the selection criteria for the different levels of the Job^Rehab^ concept, see Gutenbrunner and Schwarze 2011 [32].

The assessment was carried out by self-report of the employer and examination of the company physician. The range of MSD went from muscular tension, spine and joint diseases and others.

Further inclusion criteria assessed by the CP were work-related functional limitations, risk of losing earning capacity, anticipated employment period in the company for at least one year, and no reported intention to retire.

Access was voluntary, and participation occurred during paid work time.

### 2.3. Intervention

Information about the program was spread in the company via superiors, staff meetings, leaflets, and medical services. Enrollment and first registration took place at the company’s medical service, followed by an appointment with the CP. The CP checked the inclusion criteria for Level I (worksite intervention), and a short, functional capacity evaluation according to Isernhagen and a workplace description of the employee was conducted [36,37]. Via the use of standardized formats and communication procedures, the CP transferred all information, including recommendations regarding treatment of specific problems in the musculoskeletal system. Qualified employees were then recommended to the rehabilitation physician (RP) [35].

Depending on the job profile information of the CP, the clinical picture, and specific MSD complaints identified, individualized work-related therapy was planned by the RP.

The intervention comprised of a one week (five full days from nine to five) of intensive intervention, training and three months of twice-weekly training (one hour). It consisted of the following preventative and rehabilitative components: diagnostic and assessment, workplace-related medical and physiotherapy, workplace-related occupational therapy, workplace-related medical training therapy, occupational therapy testing at the workplace, if necessary adjustment at the workplace, health and nutrition counseling, and after-care counseling. At the end of the three months, a final consultation with the CP was provided.

During the running of the project it was integrated into the hospital’s corporate health management and monitored by a Trust Board consisting of employees' council, personnel development, and representatives of the participants’ divisions. In regular meetings, workplace problems, work conditions and current and further project developments and data security were discussed.

### 2.4. Data Collection and Measurements

Sociodemographic characteristics and information of the work divisions and conditions of the employees were assessed at baseline. The satisfaction of participants with the intervention was recorded with a selection of items of the ZUF-8 questionnaire three months later at the end of the intervention [38]. In addition, self-developed questions by the authors were used.

Satisfaction with the work condition and design, and job motivation were measured with self-design, project-specific questionnaires at t1 and t2. The Work Ability Index (WAI), a seven-part self-assessment was used to measure work ability as a primary outcome. The WAI evaluates: current ability, work ability in relation to physical and mental demands of the job, reported diagnosed diseases, estimated impairment due to health status, sick leave over the last six months, and self-prognosis of work ability in the upcoming two years and mental resources. The four outcome categories are subdivided into “poor” (7–27), “moderate” (28–36) “good” (37–43) or “excellent” (44–49) [39]. Secondary outcomes were prognosis of future employment; this was measured with the SPE-Scale, a short scale assessing the subjective prognosis of gainful employment [40]. Human resource management provided data on days of sick leave. The personal data collected were not documented in the hospital's patient systems but kept in a separate paper file.

### 2.5. Statistical Analyzes

Descriptive statistics were used to summarize the sample and to describe the main findings. Values were presented as frequencies in percent, as a mean and standard deviation (SD). The same applied to work conditions and satisfaction with the program and work conditions.

The sum scores and categories of the WAI and SPE-Scale were illustrated and described.

To compare means between pre- and post-measurement analysis of variance were carried out and standardized effect sizes (SES) were calculated. When interpreting the effect of the intervention general guidelines analog Cohen were used: small (0.2), medium (0.5) and large (0.8) [41].

Numbers of days of sick leave were compared six months before and the six months post the worksite program.

Regression models, including sociodemographic, and health and work condition related variables, were calculated to identify risk factors affecting the ability to work after intervention.

A *p*-value < 0.05 was considered to be significant.

We used Statistical Package for Social Science in Version 19.

## 3. Results

### 3.1. Sample Description

**Figure 1:**
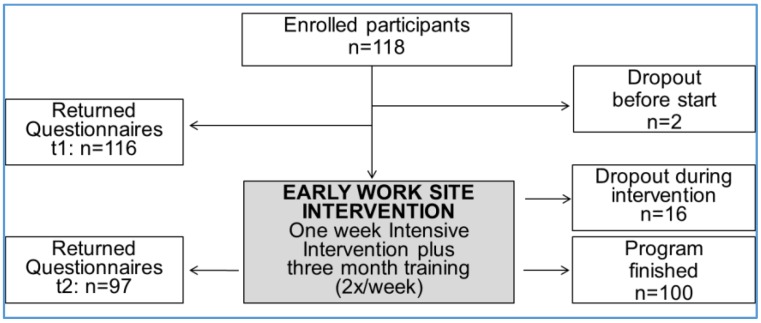
Study group: participation and dropout.

A total of 118 employees initially enrolled in the program; two dropped out before the intervention started and another 16 participants later dropped out of the program, in most cases because of acute illness. Of the remaining 100 participants, 97 completed the data for the two time points (Figure 1).

Table 1 shows sample characteristics at t1. The average age was 47 with 60.3% women (*n* = 70) and 39.7% (*n* = 46) men. In the program, 59.5% of workers had physically stressful work activity (e.g., bed central, laundry, transportation), while 40.5% were office workers (e.g., finance department, patient accounting) of the hospital.

### 3.2. Participation and Satisfaction with the Worksite Intervention

Of the 116 participants enrolled at t1, 100 workers completed the whole program (i.e., intensive week plus three-month training). Workers rated the program for its help in coping with MSD complaints at the workplace; 52.6% said it was very helpful, 39.2% said it helped some, 7.2% said it did not help, and 1% said it made things more difficult. At the end of the intervention (t2), 95.9% of the participants rated the quality of the program as very good or good, 3.1% rated it as less good and 1% rated the quality as bad. Ninety-eight percent would recommend the program to a colleague.

**Figure 2:**
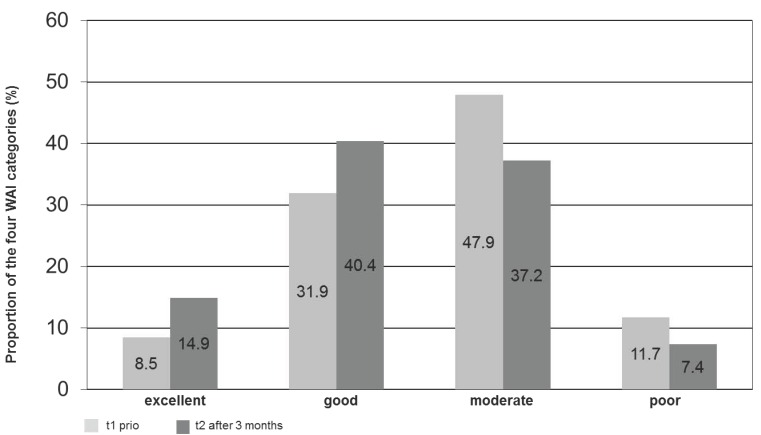
Prevalence of the WAI categories before and after worksite program (*n* = 94).

Participants’ WAI scores at t1 were good or moderate for almost 79.8% (Figure 2). Based on these findings, the WAI concept recommended interventions that promote and improve the work ability of those persons. A poor WAI score indicates that high intensive measures such as rehabilitation are necessary. For workers with excellent WAI scores, work ability should be maintained through preventative measures (e.g., recreational sports).

By means of the screening with the SPE Scale, 49.5% of the workers reported a low to very high risk of losing earning capacity.

### 3.3. Workability, Prognosis of Gainful Employment and Sickness Absence

Figure 2 shows the distribution of the four WAI categories from t1 to t2. The percentage of workers in categories with increasing workability rose, and SPE Scale data went in the same direction. The percentage of workers in the group with no risk of losing earning capacity rose from 50.5% to 55.7%. The group with a strong risk of losing earning capacity declined from 19.6% to 14.4%.

**Figure 3:**
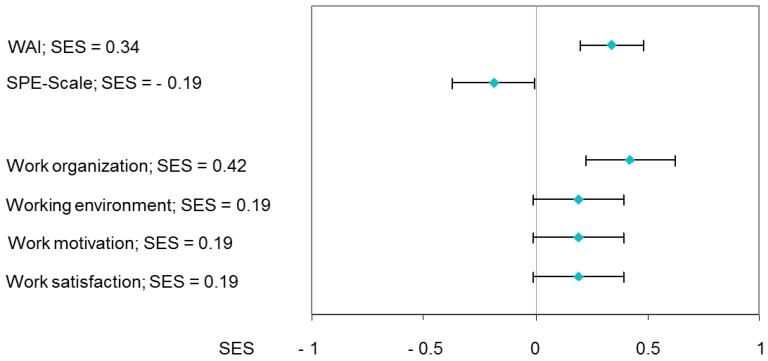
WAI (*n* = 94), SPE-scale (*n* = 97) and working conditions (*n* = 97); Standardized effect sizes (SES).

During the program, the WAI sum score improved from an average of 35.2 to 37.3 points after three months. This corresponded to an effect size of SES = 0.34, *p* < 0.001. Similarly, participation in the early worksite program had positive effects on earning capacity (SES = −0.19; *p* = 0.047) (Figure 3).

Regression analysis showed that older participants and men benefited less from participation in the worksite intervention than woman. Shift work, lower work motivation also had negative effects on the ability to work.

After six months, sick leave per participant was reduced by five days (38.5%) compared to the six-month period before the program.

## 4. Discussion

The aging workforce and increasing numbers of chronic diseases make rehabilitative health management and, specifically, the implementation of early workplace intervention increasingly necessary. Workers in the healthcare sector face special risk factors in their workplace, and musculoskeletal pain and symptoms of MSD are widely reported [9,10,11]. Literature suggests that work health programs (WHP) can increase employees’ health and productivity when designed with appropriate components, address the needs of the participants, and evaluate the program effects [42].

### 4.1. Implementation

The results of this pilot study show that the early worksite program was both practicable and successfully implemented. The program has been widely embraced, and workers were keen to participate. Dropouts, either prior to the start or while the program was being implemented, typically resulted from individuals encountering extraneous or random factors such as illness rather than lack of interest or disapproval. Owing to preparation on an organization level, potential environmental barriers were minimized effectively. A supportive management climate in the company created easy accessibility. Nevertheless, it should be mentioned that although the need of WHP for preventative and rehabilitative measures for workers with MSD in this target group is clear, almost two years of careful planning and preparation prior to the program start were necessary.

A systematic review describes the numerous factors that determine the successful implementation of preventative programs in healthcare settings [43]. This also applies to our study. As a facilitator in our study, cooperation with all involved stakeholders was identified as absolutely essential. Inclusion of members of the employees’ council and superiors played a key role, as did coordinating with the decision-makers of human resources management and the hospital board. Persuasion of all parties was needed to clear hesitancy and troubling questions. Positive evaluation results of the Job^Rehab^ program from other industrial sectors that exist were convincing [32,33]. Also, questions relating to the security of data (patient records) seemed a serious barrier, but that problem was resolved satisfactorily.

Financing the program also was a challenge, and was overcome when the management of the company decided to make the necessary investments. Loss of productivity, caused by the fact that the program would be conducted during normal working hours, was considered but deemed acceptable as it was directed to improving the health of the workers. Return of investment and long-term economic benefits were expected. Experience with the Job^Rehab^ concept in the Department of Rehabilitation Medicine initiating the program was highly advantageous. Not least, extra resources in the medical occupational service department had to be provided. The CPs had to be trained in procedures and provided extra services in screening workers. As concluded in a systematic literature research among others as reasons for insufficient reintegration, the unsatisfactory communication and cooperation between rehabilitation and occupational health physicians was seen. CDs in the present study had an objective role as mediators and acted as the connecting piece in relation to the medical interfaces in the rehabilitation process [44].

### 4.2. Need-Based Approach

Based on the screening by the CPs during the enrollment process, a group of workers most at risk of losing earning capacity due to MSD and job strain could be identified. According to WAI categories, 79.8% of the sample demonstrated the intended inclusion criteria for participation. They were found in need of preventative rehabilitative measures and were able to receive appropriate support to maintain or even to improve their work ability. These findings are somewhat higher than the results found by a working group evaluating a program similar to that described here. They showed that in “Protect Employability”, a program in rehabilitation units offered by the German pension insurance, 72.2% of the participants fell in these two WAI categories, and initial WAI scores here were 32.3. Rehabilitants with MSD in orthopedic inpatient rehabilitation units showed a significantly lower work ability (WAI = 29) [45].

A recent methodology study on the prognostic value on sickness absence confirms that the WAI can be used as a suitable instrument to identify workers at risk of prolonged sickness absence. Nevertheless, owing to low sensitivity, the authors recommend using the approach described in combination with others [46].

A critical look should be given to the 8.7% of participants with poor health who need more intensive measures such as rehabilitation as well as to the 11.7% of participants with excellent work ability. For the latter, work ability should be maintained through primary prevention such as physical activity; inclusion of these workers could be rated as an over-under or misuse of the worksite program. However, it cannot be ruled out that participants were included for other meaningful reasons in addition to the WAI screening.

However, focusing on the program’s target and intervention group in the first step seems to be more practicable. Introduction of the entire Job^Rehab^ model was not feasible. The essential structures to facilitate and coordinate further steps such as inpatient or outpatient intensive Job^Rehab^ or preventative measures such as physical activity were not yet available. Exclusion of the interested employees from the worksite program was avoided for motivational reasons.

As already recognized and implemented in the previous project, in Job^Rehab^ it is essential to provide need-based interventions. However, this has been recognized in the project group and the accompanying trust board and improvements have been tackled. Furthermore, it was obvious that the amount of psychosocial support was too little, so in the future workers should get more and differentiated measures to deal with stress in the workplace.

In a follow-up model, besides the Department of Rehabilitation Medicine, the Institute of Sports Medicine, the Department of Psychiatry, Social Psychiatry and Psychotherapy of the hospital as well as the regional German Pension were included as additional partners. In this way rehabilitation and case management as well as psychosocial measures and physical activity were provided for the workers. In conclusion, employees could use the services of the university hospital for their own health and fitness [47].

### 4.3. Outcome

Small to moderate effect sizes could be reported in terms of measures of effectiveness of work ability, prognosis of gainful employment, and work situation. Compared with the intervention “Protect employability” in the present study, the risk profiles measured with the WAI and SPE Scale also improved significantly [45]. A review on workplace interventions for preventing work disability concluded that there is moderate evidence that workplace interventions reduce sickness absence among workers with MSD, but where it is not effective is in improving health outcomes [17].

The reduction of sickness absence between 38.5% (six-month period) was found in this study. At Volkswagen Commercial Vehicle, sickness absence declined by 40.5% in the early worksite intervention group (Level I) in a six-month observation period [33]. In further long-term health economics studies, it might be of interest to study if the resources invested for the intervention compared with the cost of production lost due to sickness absence lead to a return on investment.

The results also provide some information about factors determining work ability. Work ability is associated with independent variables such as individual characteristics and workplace conditions [48]. In our study, older participants in particular took less benefit than those that were younger and men took less advantage than woman. Lower work motivation and shift work had negative effects on work ability.

### 4.4. Strengths and Limitations

Some strengths and limitations have to be considered. A selection bias cannot be excluded because an uncontrolled one-group pretest-posttest design was accomplished and a dropout analysis for responders or non-responders was not carried out. It is unclear whether or not the participants were a representative sample of all workers in the selected 11 work divisions where the program was offered first. Without a control group it is not possible to compare the outcomes with other divisions who did not receive the early worksite intervention and if the effects are due to the intervention. It became clear that instruments and assessment of work conditions, work satisfaction and health status had to be more detailed and differentiated to comprehensively understand employees’ health and workplace conditions.

Nevertheless, this study was a pilot study in manageable divisions of the hospital. It can be seen as the most appropriate design to test the feasibility for further use in a future intervention with higher sample sizes in larger divisions, especially for employees in nursing, a significant work field of the university hospital.

A strength of the study is that a multidimensional measurement was used. A synthesis out of self-reported subjective health outcome measures, work ability, indicators of the work situation and objective data was conducted. By means of a pre-/post-design, the outcome of the intervention could be made clear with widespread instruments and parameters. As a successful challenge, the engagement of the stakeholders in all phases of the study can be seen. They were constantly informed and consulted about the implementation of the interventions as well as the study process and findings. Their clinical and organizational expertise was integrated in further study designs.

## 5. Conclusions

All these results suggest that the implementation of the original Job^Rehab^ model, especially the early worksite intervention (Level 1) at a university hospital, is feasible. Currently, the worksite program is offered as a routine component in the company health management of the hospital where the authors are employed. It is, meanwhile, a core element in the comprehensive health management system “Fit for Work and Life” (FWL) for employees of the university hospital.

## Figures and Tables

**Table 1:** Sample characteristics at t1, *n* = 116.

Variables	*n* (%)
**Gender**	
Female	70 (60.3)
Men	46 (39.7)
**Age**	
Average (years, SD)	47 8.3
Groups (years)	
<46	46 (39.7)
46–50	25 (29.0)
>50	41 (35.3)
Range (years max–min)	63–20
**Occupational groups/divisions**	
Blue-collar workers (logistics, transport, kitchen, laundry)	69 (59.5)
White-collar workers (funding, finance, patient-accounting office)	47 (40.5)
**Working conditions**	
Shift work	26 (22.8)
Display screen work	51 (44.7)
Physically demanding work (high load)	34 (29.8)
Time pressure (high load)	37 (32.5)
**Work Ability Index** (*n* = 94)	
Excellent (44–49)	8 (8.5)
Good (37–43)	30 (31.9)
Moderate (28–36)	45 (47.9)
Poor (7–27)	11 (11.7)
**Prognosis of gainful employment (SPE-scale)** (*n* = 97)	
No risk	49 (50.5)
Low risk	26 (26,8)
Strong risk	19 (19.6)
Very strong risk	3 (3.1)
**Days of sick leave** During six months prior to intervention (days)	13.0

## Abbreviations

The following abbreviations are used in this manuscript:

MSD	Musculoskeletal Disorders
RP	Rehabilitation Physician
CP	Company Physician
SPE-Scale	Subjective prognosis of gainful employment
WAI	Work Ability Index
WMR	Work-Related Medical Rehabilitation
WHP	Work Health Program
GDP	Gross Domestic Product
SES	Standardized Effect Size
